# Incidence of recent HCV infection among persons seeking voluntary counselling and testing for HIV and sexually transmitted infections in Taiwan

**DOI:** 10.7448/IAS.17.4.19640

**Published:** 2014-11-02

**Authors:** Yi-Ching Su, Wen-Chun Liu, Lan-Hsin Chang, Pei-Ying Wu, Yu-Zhen Luo, Cheng-Hsin Wu, Hsin-Yun Sun, Sui-Yuan Chang, Chien-Ching Hung

**Affiliations:** 1 Internal Medicine, National Taiwan University Hospital, Taipei City, Taiwan; 2 Center for Infection Control, National Taiwan University Hospital, Taipei City, Taiwan; 3 Clinical Laboratory Sciences and Medical Biotechnology, National Taiwan University College of Medicine, Taipei City, Taiwan

## Abstract

**Introduction:**

The incidence of recent hepatitis C virus infection (HCV) infection has been noted to be increasing among men who have sex with men (MSM), especially those with HIV infection, in several resource-rich settings. In Taiwan, the incidence of recent HCV infection increased from 0 in 1994–2000, 2.29 in 2001–2005 to 10.13 per 1000 person-years of follow-up (PYFU) in 2006–2010. In this study, we aimed to estimate the incidence rate of recent HCV infection among those individuals who sought voluntary counselling and testing (VCT) service at a University Hospital.

**Methods:**

Between May 2006 and December 2013, 18,246 tests for HIV antibody were performed among 12,143 individuals at the VCT services. A total of 2157 clients without HIV or HCV infection at baseline were included for estimation of incidence rate of recent HCV infection. Antibodies to HCV were determined with a third-generation enzyme immunoassay. A nested case-control study with four matched controls without HCV seroconversion for one HCV seroconverter was conducted to investigate the factors associated with recent HCV infection. Phylogenetic analysis was performed among the HCV strains obtained from VCT clients and patients coinfected with HIV and HCV between 2006 and 2013.

**Results:**

During the study period, 2157 clients received a total of 8260 tests. The HCV seroprevalence at baseline was 0.3%. Of the 2150 HCV-negative clients who contributed 5074.99 PYFU, 17 developed HCV seroconversion (incidence rate, 3.35 per 1000 PYFU; 95% CI, 1.76–4.94); the rate increased from 2.28 per 1000 PYFU (95% CI, 0.05–4.51) in 2006–2009, to 3.33 per 1000 PYFU (95% CI, 0.86–5.80) in 2010–2011, to 4.94 per 1000 PYFU (95% CI, 0.99–8.99) in 2012–2013. In case-control study, HCV seroconverters were more likely to have HIV-infected partners, recent syphilis and a Rapid Plasma Reagin (RPR) titre of 4 or greater. In multivariate analysis, having HIV-infected partners remained as the only independent associated factors with HCV seroconversion (AOR, 6.931; 95% CI, 1.064–45.163). Phylogenetic analysis revealed transmission pairs and clusters, with most clustered sequences derived from MSM.

**Conclusions:**

Similar to the observation among HIV-infected patients who are not IDUs, increasing trends of recent HCV infection also occur among the individuals who sought VCT services in Taiwan. Having HIV-infected partners is independently associated with recent HCV seroconversion.

